# Recent Developments in Positron Emission Tomography Tracers for Proteinopathies Imaging in Dementia

**DOI:** 10.3389/fnagi.2021.751897

**Published:** 2022-01-03

**Authors:** Ruiqing Ni, Roger M. Nitsch

**Affiliations:** ^1^Institute for Regenerative Medicine, University of Zurich, Zurich, Switzerland; ^2^Institute for Biomedical Engineering, ETH & University of Zurich, Zurich, Switzerland

**Keywords:** amyloid-β, tau, α-synclein, positron emission tomography (PET), Alzheimer’s disease, Parkinson’s disease, Lewy bodies, frontotemporal dementia (FTD)

## Abstract

An early detection and intervention for dementia represent tremendous unmet clinical needs and priorities in society. A shared feature of neurodegenerative diseases causing dementia is the abnormal accumulation and spreading of pathological protein aggregates, which affect the selective vulnerable circuit in a disease-specific pattern. The advancement in positron emission tomography (PET) biomarkers has accelerated the understanding of the disease mechanism and development of therapeutics for Alzheimer’s disease and Parkinson’s disease. The clinical utility of amyloid-β PET and the clinical validity of tau PET as diagnostic biomarker for Alzheimer’s disease continuum have been demonstrated. The inclusion of biomarkers in the diagnostic criteria has introduced a paradigm shift that facilitated the early and differential disease diagnosis and impacted on the clinical management. Application of disease-modifying therapy likely requires screening of patients with molecular evidence of pathological accumulation and monitoring of treatment effect assisted with biomarkers. There is currently still a gap in specific 4-repeat tau imaging probes for 4-repeat tauopathies and α-synuclein imaging probes for Parkinson’s disease and dementia with Lewy body. In this review, we focused on recent development in molecular imaging biomarkers for assisting the early diagnosis of proteinopathies (i.e., amyloid-β, tau, and α-synuclein) in dementia and discussed future perspectives.

## Introduction

Today, nearly 50 million worldwide live with dementia. This number is projected to reach 152 million in 2050 as the population ages (Bhatt et al., 2019; Cummings et al., 2021b). An early detection and intervention for dementia represent tremendous unmet clinical needs and priorities in the aging societies. Neurodegenerative diseases, including Alzheimer’s disease (AD), Parkinson’s disease (PD), frontotemporal dementia (FTD), and dementia with Lewy bodies (DLB), are the most common causes of dementia. In these diseases, the abnormal accumulation of aggregates of the pathological protein activates a cascade of biochemical changes and affects the selective vulnerable circuit in a disease-specific pattern (Pievani et al., 2014; Bang et al., 2015; Goedert, 2015; De Strooper and Karran, 2016; Jucker and Walker, 2018; Soto and Pritzkow, 2018; Park et al., 2020). AD is pathologically hallmarked by amyloid-β (Aβ) plaque, neurofibrillary tangle (NFT), and neuronal loss (Knopman et al., 2021; Scheltens et al., 2021). Clinically, AD is characterized by the progressive loss of memory and cognitive functions, gradually affecting the daily life of patients. *In vivo* imaging studies in AD have shown that molecular changes in the brain precede the occurrence of clinical symptoms of cognitive decline by a long period, up to 15 years (Palmqvist et al., 2021). FTD includes a spectrum of tauopathy diseases, including corticobasal disease (CBD), progressive supranuclear palsy (PSP), and Pick’s disease (Spillantini and Goedert, 2013), clinically characterized by progressive executive, behavioral, or language dysfunctions depending on the disease types (Spillantini and Goedert, 2013). DLB, PD, and multiple system atrophy (MSA) are pathologically characterized by the appearance of Lewy bodies and Lewy neurites, composed of aggregated α-synuclein fibrils (Fares et al., 2021). The loss of dopaminergic neurons in the substantia nigra is the major pathological hallmark of PD (Poewe et al., 2017). The clinical diagnosis of PD is based on the motor dysfunction symptoms, including bradykinesia, rigidity, and resting tremor due to the nigrostriatal degeneration. Overlapping clinical symptoms and comorbidities in different diseases impose challenges on the accurate disease diagnosis, especially at a prodromal or early disease stage (Irwin et al., 2013). For example, the clinical symptoms in PD overlap with that in MSA and PSP to a certain extent (Politis, 2014). AD overlaps in the symptom or in pathological features with vascular dementia, FTD, and DLB (Knopman et al., 2021). Thus, a highly specific biomarker or combinations of biomarkers for increasing the diagnostic accuracy and enabling optimal treatment strategy are highly desired. In this review, we focused on the recent developments in positron emission tomography (PET) tracers for the detection of proteinopathies (i.e., Aβ, tau, and α-synuclein) in neurodegenerative diseases.

## Positron Emission Tomography for Proteinopathies in Neurodegenerative Diseases

The advances in molecular imaging using PET, structural and functional imaging using magnetic resonance imaging, cerebrospinal fluid assays for detecting disease pathological hallmarks have facilitated the early and differential diagnosis and clinical management in AD, as well as the understanding of the disease mechanism and development of therapeutics (Sevigny et al., 2016; Crunkhorn, 2017; Boxer et al., 2019; Rabinovici et al., 2019; Chételat et al., 2021; Hansson, 2021). [^18^F]fluorodeoxyglucose (FDG)-PET has been used for detecting the cerebral glucose hypometabolism in disease-specific brain regions in patients with AD, FTD (Foster et al., 2007; Chételat et al., 2020), and idiopathic PD and atypical parkinsonism associated with dementia improving the diagnostic accuracy (Walker et al., 2018). There is a rapid advancement in recent 20 years in the development of specific PET tracers for pathological proteinopathies, neuroinflammation, and synaptic density markers in neurodegenerative diseases. Several prerequisites need to be fulfilled for an ideal PET tracer, including low molecular weight, sensitivity, specificity (i.e., low off-target binding), high affinity, moderate lipophilicity, solubility, blood-brain barrier entrance (i.e., sufficient brain uptake), reversible binding property, and pharmacokinetics, as well as no radiolabeled metabolites in the brain (Pike, 2009).

### Amyloid-β Imaging

Amyloid-β is produced by proteolytic processing of the amyloid precursor protein on the neurons and glial cells. An imbalance between the production and clearance of Aβ leads to its abnormal cerebral accumulation (i.e., accumulation of oligomers, protofibrils, fibrils, and amyloid plaques), which plays a central role in the pathogenesis of AD both in animal models and in patients (Lesné et al., 2006; Haass and Selkoe, 2007; Lambert et al., 2007; Shankar et al., 2008; Selkoe and Hardy, 2016). The spread of Aβ follows a specific pattern, starting from neocortical regions to regions that receive neuronal projections and later to subcortical regions such as the striatum and the cerebellum (Thal et al., 2002). Using amyloid PET imaging combined with a functional MRI, the earliest accumulation of Aβ is found within the default mode network and, concurrently, affects the brain connectivity (Altmann et al., 2015; Palmqvist et al., 2017; Sepulcre et al., 2017; Hanseeuw et al., 2019; Rabinovici et al., 2019; Vogel et al., 2020). Amyloid PET tracers detect the β-sheet structures and are mainly benzothiazole and benzoxazole derivatives (Table 1; Klunk et al., 2004; Rowe et al., 2008; Furukawa et al., 2009; Nelissen et al., 2009; Nyberg et al., 2009; Hostetler et al., 2011; Cselenyi et al., 2012; Rodriguez-Vieitez et al., 2015; Sehlin et al., 2016; Grimmer et al., 2020; Meier et al., 2021; Ni, 2021; Ni et al., 2021). Among these tracers, three have been approved by Food and Drug Administration (FDA) and European Medicines Agency for clinical usage, namely, [^18^F]flutemetamol (Vizamyl), [^18^F]florbetapir (Amyvid), and [^18^F]florbetaben (Neuraceq) (Clark et al., 2011; Curtis et al., 2015; Sabri et al., 2015). PET studies using amyloid probes [^11^C]PiB, [^18^F]flutemetamol, [^18^F]florbetapir, [^18^F]florbetaben, and [^18^F]flutafuranol (AZD4694) have demonstrated higher cortical fibrillar Aβ loads in patients with mild cognitive impairment and AD compared with those in healthy controls (Klunk et al., 2005; Clark et al., 2011; Curtis et al., 2015; Sabri et al., 2015; Jack et al., 2018; Wolk et al., 2018). A robust *in vivo* congruence between aforementioned tracers and an *in vivo* postmortem correlation have been demonstrated in the human brain (Villemagne et al., 2012; Rowe et al., 2013; Ni et al., 2017, 2021; Su et al., 2019; Ikonomovic et al., 2020). It is noted that Aβ deposits are detected in the non-demented control, with the incidence associating with increasing age (Pike et al., 2007). In the context of a structured 5-phase development framework, amyloid PET using aforementioned tracers has already reached the clinical utility phase (Cotta Ramusino et al., 2021). It has been established as a pathological biomarker for early and differential diagnosis of AD continuum based on both the international working group and the National Institute on Aging-Alzheimer’s Association research AT(N) framework (Frisoni et al., 2017; Jack et al., 2018; Dubois et al., 2021) and recently proposed ATX(N) conceptual framework (Hampel et al., 2021). To further ensure a standardized outcome measure, and to reduce the disagreement across amyloid-PET imaging, the readouts have been converted into Centiloid units (Klunk et al., 2015). This is based on the normalization of the data from [^18^F] amyloid tracers relative to [^11^C]PiB, with young controls as zero and averages from typical patients with mild-moderate AD as 100 (Klunk et al., 2015). Recent probes with an improved binding specificity and lower bone uptake, such as [^18^F]FIBT, [^18^F]FACT, and [^18^F]D15FSP (Ito et al., 2014; Grimmer et al., 2020; Xiao et al., 2021), or that detect diffuse amyloid, such as benzoselenazole derivative [^18^F]fluselenamyl, have been developed (Sundaram et al., 2016). Antibody-based PET/single-photon emission computed tomography (SPECT) tracers [^124^I]RmAb158-scFv8D3 and [^124^I]8D3-F(ab’)2-h158 have showed sufficient blood-brain barrier entrance, by conjugating to transferrin receptor antibodies, in several transgenic mouse models of amyloidosis (Sehlin et al., 2016; Meier et al., 2021).

**TABLE 1:** Positron emission tomography and SPECT imaging tracers for detecting proteinopathies; *in vivo* evaluation in human and in animal models.

**Target**	**Structure**	**Ligands**	**Human**	**Animal**
Aβ	Benzothiazole	[^11^C]PiB	(Klunk et al., 2004)	(Klunk et al., 2005)
		[^18^F]florbetapir	(Clark et al., 2011)	(Poisnel et al., 2012)
		[^18^F]florbetaben	(Rowe et al., 2008)	(Rominger et al., 2013)
		[^11^C]AZD2184	(Nyberg et al., 2009)	(Rodriguez-Vieitez et al., 2015)
		[^18^F]flutafuranol	(Cselenyi et al., 2012)	(Parent et al., 2017)
		[^18^F]flutemetamol	(Thal et al., 2015)	(Snellman et al., 2012)
		[^18^F]FIBT	(Grimmer et al., 2020)	(Yousefi et al., 2015)
	Benzofuran	[^18^F]FPYBF-2	(Higashi et al., 2018)	(Cheng et al., 2010)
	Benzoxazole	[^18^F]FACT	(Ito et al., 2014)	(Furumoto et al., 2013)
		[^11^C]BF-227	(Kudo et al., 2007)	(Kudo et al., 2007)
		[^18^F]MK-3328		(Hostetler et al., 2011)
		[^18^F]AD-269		(Hostetler et al., 2011)
	Benzoselenazole	[^18^F]fluselenamyl		(Sundaram et al., 2016)
	Antibody Antibody	[^124^I]RmAb158-scFv8D3		(Meier et al., 2021)
		[^124^I]8D3-F(ab’)2-h158		(Sehlin et al., 2016)
Tau	Quinoline	[^18^F]THK-5105	(Okamura et al., 2014)	(Okamura et al., 2013)
		[^18^F]THK-5117	(Harada et al., 2015)	(Brendel et al., 2016)
		[^11^C]THK-5351	(Harada et al., 2016)	(Harada et al., 2016)
		[^11^C]THK-523	(Fodero-Tavoletti et al., 2011)	(Fodero-Tavoletti et al., 2011)
	Pyridinyl-butadienyl-benzothiazole	[^11^C]PBB3	(Maruyama et al., 2013)	(Maruyama et al., 2013)
		[^18^F]APN-1607 (PM-PBB3)	(Tagai et al., 2020)	(Tagai et al., 2020)
	Benzimidazole pyridine	[^18^F]flortaucipir	(Fleisher et al., 2020)	(Brendel et al., 2018)
		[^18^F]PI2620	(Mueller et al., 2019)	
		[^18^F]RO948	(Kuwabara et al., 2018)	
		[^18^F]GTP1	(Sanabria Bohórquez et al., 2019)	
	Naphtylethylidene	[^18^F]FDDNP	(Kepe et al., 2006)	(Teng et al., 2011)
		[^18^F]MK6240	(Hostetler et al., 2016)	
		[^18^F]JNJ64326067	(Schmidt et al., 2020)	(Rombouts et al., 2019)
		[^18^F]JNJ64349311		(Declercq et al., 2017)
	Pyridinyl-indole	[^18^F]CBD-2115 (4R-tau)		(Lindberg et al., 2021)
	Pyridoimidazopyridine	[^123^I]PIP-NHMe		(Watanabe et al., 2021)
α-synuclein	Pyridinyl-butadienyl-benzothiazole	[^18^F]C05-05, C-05-01		(Miranda-Azpiazu et al., 2020; Ono et al., 2020)
		[^11^C]PBB3	(Perez-Soriano et al., 2017)	(Miranda-Azpiazu et al., 2020)
	Benzoxazoles	[^18^F]BF-227, BF-227-like	(Kikuchi et al., 2010)	(Levigoureux et al., 2014)
		[^18^F]4FBox, [^18^F]2FBox		(Verdurand et al., 2018)
	Diarylbithiazole	[^18^F]FS3 (DABTA-11)	(Hooshyar Yousefi et al., 2019)	(Aboagye and Kraeber-Bodéré, 2017)
		[^18^F]DABTA-7, -8		(Uzuegbunam et al., 2020)
		[^18^F]ACI-Cpd-AE, [^18^F]ACI-12589	(Capotosti et al., 2020)	
	Indolinone	[^18^F]WC-58a		(Chu et al., 2015)
		XW-01-11, XW-01-04		(Sun et al., 2021)
	Benzofuranone	[^3^H]Tg-190b		(Ferrie et al., 2020)
		[^3^H]BF2846		(Ferrie et al., 2020)
	Phenothiazine	[^125^I]SIL23, [^18^F]SIL26		(Bagchi et al., 2013)
	Diphenyl	[^125^I]IDP-4		(Ono et al., 2016)
	Bisquinoline	[^18^F]BQ2		(Kaide et al., 2020)
	Diphenylpyrazole	[^11^C]MODAG-001		(Kuebler et al., 2021)
		[^11^C]anle253b		(Maurer et al., 2020)

### Tau Imaging

**FIGURE 1:**
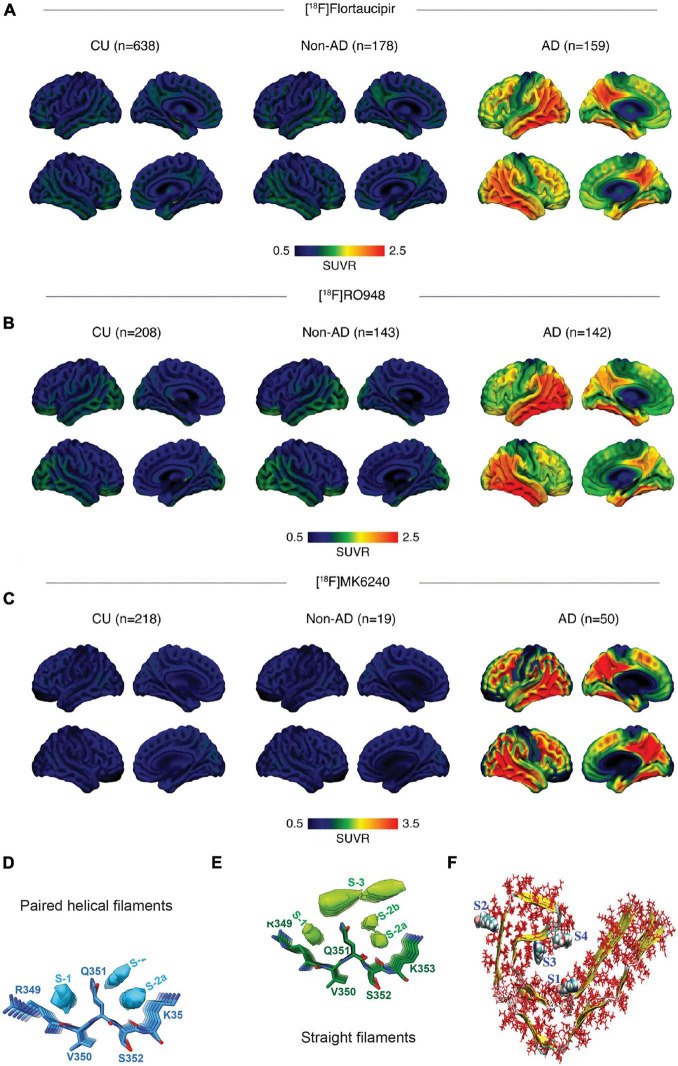
*In vivo* and postmortem comparison of tau imaging probes in the human brain **(A–C)** multicenter comparison of positron emission tomography (PET) imaging using [^18^F]flortaucipir, [^18^F]RO948, and [^18^F]MK6240, standardized uptake value ratios (SUVRs) across all participants within diagnostic groups; non-demented controls (CU), Alzheimer’s disease (AD); reproduced from Leuzy et al. (2021) with permission from Springer Nature; **(D,E)** binding of APN-1607 (PM-PBB3) to tau-paired helical filaments and straight filaments is based on cryo-EM, top views and side views of the extra densities in the PM-PBB3 binding sites of paired helical filaments **(D)** and straight filaments **(E)** maps. The models of PM-PBB3 are shown near these extra densities at the same scale. Reproduced from Shi et al. (2021a) with permission from Springer Nature; and **(F)** various high-affinity binding sites of tau protofibril. The sites 1, 3, and 4 are termed core sites as they are buried inside the fibril, whereas site 2 is termed a surface site as it is exposed to a greater amount of solvent molecules. Reproduced from Murugan et al. (2018) with permission from American Chemical Society.

Microtubule-associated tau protein (MAPT) is located inside the neurons and is produced by alternative splicing from *MAPT* gene on chromosome 17. Tau has important physiological functions in regulating the axonal transport and neurite outgrowth and maintaining the microtubule stability (Chang et al., 2021). In AD brain, both 3-repeat (3R) and 4-repeat (4R) tau are presented, as 4R tau in CBD and PSP brain and 3R tau in Pick’s disease brain (Iqbal et al., 2010; Shi et al., 2021b). Tau is abnormally hyperphosphorylated forming oligomer, fibrils, and NFTs (Iqbal et al., 2010; Spillantini and Goedert, 2013). In the AD brain, tangles accumulate first in the entorhinal cortex (Braak and Braak, 1991) and, subsequently, spread from the entorhinal cortex to the hippocampus and neocortex *via* neuronal projection, leading to the disruption of the microtubule stability and cell death (Holmes et al., 2014; Franzmeier et al., 2019). MRI readouts of neurodegeneration and functional network alterations associate with tau and Aβ accumulation detected by PET in patients with mild cognitive impairment and AD (Jacobs et al., 2018; Franzmeier et al., 2019; La Joie et al., 2020; Vogel et al., 2020). Several tau tracers have been developed, including first-generation [^18^F]flortaucipir (Johnson et al., 2016), [^11^C]PBB3 (Maruyama et al., 2013), [^11^C]THK-523 (Fodero-Tavoletti et al., 2011), [^18^F]THK-5117 (Okamura et al., 2013), [^18^F]THK-5105 (Okamura et al., 2014), and [^18^F]THK-5351 (Harada et al., 2016) and second-generation [^18^F]MK6240 (Lohith et al., 2019), [^18^F]RO948 (Leuzy et al., 2020), [^18^F]PI2620 (Mueller et al., 2019), [^18^F]PM-PBB3 (APN-1607) (Tagai et al., 2020), [^18^F]JNJ-64326067 (Schmidt et al., 2020), and [^18^F]GTP1 (Sanabria Bohórquez et al., 2019). In the context of a structured 5-phase development framework of biomarkers for AD, the first- and second-generation tau PET tracers are currently considered at the clinical validity phase (Bischof et al., 2021; Chiotis et al., 2021; Wolters et al., 2021). Among these tracers, [^18^F]flortaucipir (Tauvid) has been approved by FDA for imaging tauopathy in patients with cognitive impairments undergoing evaluation for AD. [^18^F]flortaucipir has been used in clinical trials to monitor the development of regional tauopathy in patients with AD during immunotherapy, targeting Aβ (Cummings et al., 2021a; Knopman et al., 2021). The off-target binding to monoamine oxidase-B (MAO-B) and in the choroid plexus was reported with the first-generation tracers, namely, [^18^F]flortaucipir, [^18^F]THK-5117, and (S)-[^18^F]THK-5117 (Sander et al., 2016; Lemoine et al., 2018; Wren et al., 2018; Murugan et al., 2019). In addition, Hansen et al. showed a decrease in the [^18^F]flortaucipir binding to neuromelanin in the midbrain of patients with PD compared with controls, reflecting the loss of pigmented neurons in the substantial nigra (Hansen et al., 2016). With the improved design, no clear off-target binding was reported for the second-generation tau imaging probe in the choroid plexus *in vivo* (Mueller et al., 2019; Sanabria Bohórquez et al., 2019; Leuzy et al., 2020; Pascoal et al., 2020; Schmidt et al., 2020; Tagai et al., 2020) or to MAO-B in postmortem investigations (Yap et al., 2021). Leuzy et al. (2021) reported a multicenter comparison study and suggested that a common temporal lobe region of interest and cut-off can be used for the differential diagnosis of patients with dementia with [^18^F]flortaucipir, [^18^F]RO948, and [^18^F]MK6240 tau PET (Figures 1A–C). For the primary tauopathy diseases, Kroth et al. (2019) and Brendel et al. (2020) showed that [^18^F]PI2620 showed a higher uptake in the basal ganglia of patients with PSP compared with that in controls by PET, with a high specificity in the brain from patients with PSP at the postmortem. Tagai et al. (2020) showed a distinct tau distribution pattern using PET with [^18^F]PM-PBB3 in patients with PSP in the basal ganglia and patients with AD in the cortex and hippocampus compared with that in control. Yap et al. (2021) recently compared the second-generation probes PI2620, RO948, MK6240, and JNJ-64326067 in postmortem brain tissues from patients with AD, PSP, CBD, and Pick’s disease by using autoradiography and immunohistochemistry and demonstrated that these tracers could detect cortical paired-helical-filament tau and a lower binding to cortical inclusions of primary tauopathies.

There is currently a lack of tracers specific for 4R tau. Animal models, such as P301L and P301S, that recapitulate pathological features of 4R tauopathy have been developed with mutations in the *MAPT* gene, (Lewis et al., 2000; Gotz et al., 2001; SantaCruz et al., 2005; Spires et al., 2006), as well as hTau and knock in animal models (Saito et al., 2019; Hosokawa et al., 2021). The *in vivo* imaging of tau has been demonstrated in animal models using [^18^F]APN-1607, [^11^C]PBB3, [^11^C]mPBB5, [^18^F]THK5117, and [^18^F]JNJ64349311 (Maruyama et al., 2013; Brendel et al., 2016, 2018; Declercq et al., 2017; Ishikawa et al., 2018; Ni et al., 2018; Tagai et al., 2020; Vagenknecht et al., 2021), as well as SPECT using [^123^I]PIP-NHMe (Watanabe et al., 2021). It is noted that [^18^F]flortaucipir did not detect tau in the rTg4510 (P301L) 4R-tau mouse model (Marquié et al., 2015; Ni et al., 2018). More recently, tracer pyridinyl-indole derivative [^18^F]CBD-2115 has shown 4R-specific detection and promising brain uptake in mouse, rat, as well as non-human primate (Lindberg et al., 2021).

### α-Synuclein Imaging

**FIGURE 2:**
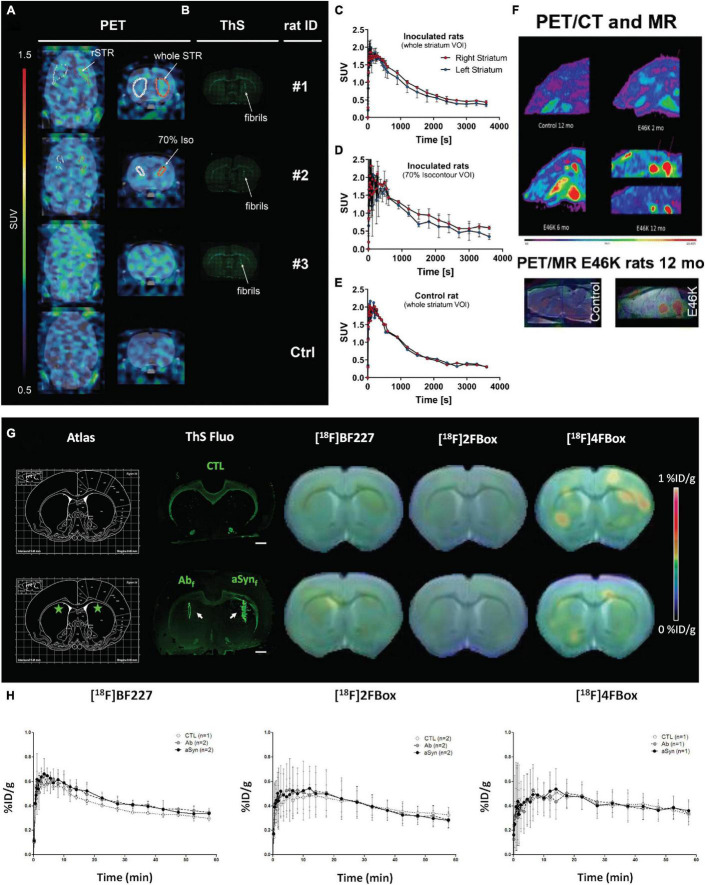
*In vivo* α-synuclein imaging in animal models. **(A–E)** *In vivo* binding of (d_3_)-[^11^C]MODAG-001 in α-synuclein-inoculated rats. Coronal and transversal PET images (2.5–60 min) **(A)**. Images show increased tracer accumulation in the α-synuclein fibril-inoculated right striatum compared with the vehicle-injected contralateral striatum. Thioflavin-S staining **(B)** indicated α-synuclein fibrils in the right striatum of fibril-inoculated rats **(B)**. **(C–E)** Time activity curves of (d_3_)-[^11^C]MODAG-001 higher signal in the right (α-synuclein injected) than left (vehicle injected) striatum; α-SYN, α-synuclein; rSTR, right striatum; ThS, thioflavin S; Ctrl, control; SUV, standardized uptake value, DVR-1, distribution volume ratio-1; VOI, voxel of interest; Reproduced from Kuebler et al. (2021) with permission from Springer Nature. **(F)** [^18^F]DABTA-11 PET images in E46K rats show accumulation of the tracer in the medulla oblongata. The accumulation is apparent even at 2 months of age and is more prominent at 6 and 12 months of age with detectable uptake in the substantia nigra. PET/MRI and rat brain atlas confirm the regional uptake of the tracer. Reproduced from Yousefi et al. (2016) and Aboagye and Kraeber-Bodéré (2017) with permission from Springer Nature. **(G,H)** small-animal PET imaging with [^18^F]BF227, [^18^F]2FBox, and [^18^F]4FBox in control and fibril-injected rats. **(G)** Summed PET images were coregistered with CT images, and the radioactivity index was reflected by a color scale representing %ID/g. ThS fluorescence staining of Aβ42 and α-syn fibrils injected in the striata is presented (white arrows), with the corresponding stereotaxic brain atlas region (green stars representing injection sites). Scale bar represents 1 mm on ThS fluorescence staining. **(H)** Time activity curves (expressed in %ID/g over time) for each radiotracer are presented. Values (mean ± SD) were extracted from the striata regions based on an in-house-made MRI atlas that was coregistered to PET-CT images. Reproduced from Verdurand et al. (2018) with permission from American Chemical Society.

A highly desired, but so far unmet clinical need, is the *in vivo* visualization of the cerebral accumulation of α-synuclein in individuals with α-synucleinopathies, including patients with PD, DLB, and MSA (Poewe et al., 2017; Attems et al., 2021). The α-synuclein inclusions are mainly located in the presynaptic neurons in PD and DLB, while in oligodendroglial cells in MSA. Dopamine transporter imaging using [^18^F]DOPA PET or [^123^I]FP-CIT SPECT (DAT scan) is commonly utilized to visualize dopaminergic deficits in PD (Maltais et al., 2020). [^18^F]FDG PET visualizes the cerebral glucose metabolism in DLB- and MSA-related patterns (Niethammer and Eidelberg, 2012) and differentiates between patients with classical PD, atypical parkinsonian syndromes, and healthy control. The accumulation of misfolded α-synuclein occurs at Braak stage 1 in PD, preceding the loss of dopaminergic neurons that occurs at Braak stage 4 in the substantia nigra (Politis, 2014). Thus, the development of PET imaging for α-synuclein deposits would enable an early diagnosis of α-synucleinopathies and facilitate clinical trials targeting α-synuclein (Poewe et al., 2017; Attems et al., 2021). A few structures and imaging tracers for α-synuclein, such as BF-227 alike compounds, [^11^C]PBB3, [^18^F]C05-05, [^11^C]MODAG-001, [^18^F]FS3 (or DABTA-11, –7, –8), [^18^F]ACI-Cpd-AE, [^18^F]ACI-12589, [^18^F]4FBox, and [^18^F]2FBox, have been identified and evaluated *in vitro* (Yu et al., 2012; Bagchi et al., 2013; Koga et al., 2017; Verdurand et al., 2018; Hooshyar Yousefi et al., 2019; Capotosti et al., 2020; Ono et al., 2020; Uzuegbunam et al., 2021; Table 1). Many of the current α-synuclein PET tracers display insufficient selectivity, inadequate brain uptake, or pharmacokinetics. Among these, only four tracers have so far been evaluated in human subjects with α-synucleinopathy, namely, (1) *in vivo* PET using [^11^C]BF-227 PET in patients with MSA showed higher brain accumulation compared with healthy control (Kikuchi et al., 2010). However, BF-227 also detects Aβ pathology and is insensitive to α-synuclein in brain from α-synuclein transgenic mouse model (Levigoureux et al., 2014). (2) *In vivo* PET using [^11^C]PBB3 has been performed in patients with MSA. However, the signal source was inconclusive due to the comorbidity in the brain (Perez-Soriano et al., 2017). [^11^C]PBB3 showed a lower affinity and selectivity binding to α-synuclein fibrils compared with tau fibrils *in vitro*. Given the nanomolar concentration of [^11^C]PBB3 in *in vivo* PET, α-synuclein pathology is likely below the detection threshold (Koga et al., 2017); (3) [^18^F]FS3 showed nanomolar affinity to α-synuclein fibrils (around 100-folds selectivity over Aβ and tau fibrils), brain uptake in human, as well as in the medulla oblongata of E46K α-synuclein rat model (Yousefi et al., 2016; Aboagye and Kraeber-Bodéré, 2017; Hooshyar Yousefi et al., 2019; Figure 2F); and (4) [^18^F]-ACI-Cpd-AE demonstrated a fast brain uptake, low non-specific binding, rapid metabolism, and 10% higher relative standard uptake value in the substantia nigra of patients with PD compared with those in healthy controls (Capotosti et al., 2020).

Several new α-synuclein probes of different scaffolds have been reported recently with *in vitro*/*in vivo* evaluation in animal models. Verdurand et al. (2018) reported two new probes [^18^F]4FBo and [^18^F]2FBox that bind to α-synuclein and Aβ fibrils that show sufficient brain uptake in a rat model but not in a mouse model with α-synucleinopathy (Figures 2G,H). Kaide et al. (2020) developed a bisquinoline derivative [^18^F]BQ2 and showed a moderate brain uptake (i.e., 1.59% ID/g at 2 min and 1.35% ID/g at 60 min post injection) in the brain of a mouse model. Maurer et al. (2020) reported that diphenyl pyrazoles derivative [^11^C]anle253b, based on α-synuclein oligomer modulator anle138b (Wagner et al., 2013; Wegrzynowicz et al., 2019), exhibited a good penetration in the blood-brain barrier, brain uptake, and low background binding to the non-pathological brain. Kuebler et al. (2021) reported that diphenyl pyrazole derivative [^11^C]MODAG-001 showed a high-affinity binding to α-synuclein (i.e., 0.6 nM, 30-fold higher than to tau and Aβ fibrils) and a sufficient brain uptake in α-synuclein-inoculated rats (Figures 2A–E). Ono et al. recently reported that [^18^F]C-05-05, a compound developed based on the PBB3 structure, showed specific detection of ps129 antibody-positive phosphorylated α-synuclein in a mouse model, as well as in the non-human primate (Ono et al., 2020). In addition, several recent probes [^18^F]WC-58a (Chu et al., 2015), XW01-04, XW01-64 (Sun et al., 2021), [^3^H]BF2846, and [^3^H]Tg-190b (Ferrie et al., 2020) demonstrated > 30-fold selectivity to α-synuclein over Aβ (*in silico* and *in vitro* binding to fibrils) and in autoradiograph/staining in postmortem brain tissues.

## Discussion

The advances in PET detection of disease-specific pathological proteinopathy have facilitated the personalized and timely diagnosis of dementia and offers a window for therapeutic intervention (Hansson, 2021). The integration of PET imaging, assays of cerebrospinal fluid, MRI biomarkers, and forthcoming blood tests further increases the diagnostic power in early and differential diagnosis (Altomare et al., 2021; Cullen et al., 2021; Palmqvist et al., 2021). The application of new disease-modifying treatment such as immunotherapy will likely require screening in prodromal patients for pathological evidence, e.g., cerebral Aβ, tau, or α-synuclein accumulation, and monitoring of treatment effects (Sevigny et al., 2016; Boxer et al., 2019; Rabinovici et al., 2019). In addition, proteinopathy imaging combined with PET for synaptic loss, mitochondria dysfunction, and neuroinflammation (e.g., astrocytosis and microgliosis) enables a more comprehensive understanding of the mechanism underlying neurodegeneration associated with proteinopathies (Calsolaro et al., 2021; Pascoal et al., 2021; Zhou R. et al., 2021).

Structural variations in Aβ fibrils may contribute to variations in the disease onset and the progression rate of AD. The cryo-EM study has shown polymorphism of Aβ fibrils from the AD brain tissue (Kollmer et al., 2019). The *in vivo* imaging and postmortem studies have demonstrated different detection patterns of Aβ conformational variants in different autosomal-dominant AD (Schöll et al., 2012; Ni et al., 2017; Chen et al., 2021). *In silico* studies have implied six binding sites on Aβ fibrils, and amyloid tracers of different structures detect different sites on Aβ fibrils or conformations (Murugan et al., 2016; Kuang et al., 2019). Tau molecular diversity and posttranslational modification are important contributors for the clinical heterogeneity in patients with AD (Dujardin et al., 2020). Four trajectories of diverse tau deposition pattern have been identified in the AD brain (Vogel et al., 2021). Shi et al. recently proposed a structure-based classification of tauopathy diseases underlined the tau strain heterogeneity and challenge in developing imaging probes specific for certain tau strain (Shi et al., 2021b). *In vivo* and *postmortem* comparative studies using different tau tracers indicated even more divergent patterns among tracers in primary tauopathies than in AD (Ono et al., 2017; Schonhaut et al., 2017; Chen et al., 2018; Endo et al., 2019; Ikeda et al., 2019; Leuzy et al., 2019; Arakhamia et al., 2020; Brendel et al., 2020; Tagai et al., 2020; Yap et al., 2021). Several recent *in silico* modeling studies suggest four binding sites on AD tau and highlighted the heterogeneity among probes binding to different tau strains: For the first-generation tracers, MK6240 and flortaucipir bind only to major binding site 1, while THK5351 binds to site 1 and 3, and PBB3 detects all four binding sites. For the second-generation tracers, PI2620, CBD-2115, and PM-PBB3 showed higher binding affinities to CBD tau compared with the 3R/4R tracer MK6240, and CBD-2115 and PM-PBB3 demonstrated higher binding affinities to AD tau compared with PI2620 (Figure 1F; Murugan et al., 2018, 2021; Kuang et al., 2020; Zhou Y. et al., 2021). Recent study reported that PM-PBB3 showed similar binding sites in cryo-EM study toward tau filaments from AD, posterior cortical atrophy, and primary age-related tauopathy (Figures 1D,E; Fitzpatrick et al., 2017; Shi et al., 2021a). Further investigations are anticipated for elucidating the tracers binding with cryo-EM structures of tau filaments from CBD and PSP and for rational designing of disease (strain) specific to develop tracers with an increased specificity and binding activity (Fitzpatrick et al., 2017; Zhang et al., 2020). The challenges of α-synuclein imaging stem from the intracellular location of α-synuclein inclusions, distinct α-synuclein strains, presence across different neurodegenerative diseases, and difficulty in finding a tracer with selectivity to α-synuclein over Aβ and tau fibrils (Yamasaki et al., 2019; Berg et al., 2021). Moreover, the cryo-EM structures of α-synuclein filaments from the brains of patients with MSA differ from *in vitro* recombinant α-synuclein fibrils (Schweighauser et al., 2020). The different α-synuclein strains contribute to the disease heterogeneity in animal models and in patients (Holec and Woerman, 2021). Klingstedt et al. (2019) and Shahnawaz et al. (2020) demonstrated a differential binding of a fluorescence luminescent-conjugated oligothiophenes probe to α-synuclein fibrils derived from patients with MSA with that from patients with PD.

Further high-throughput screening and structure-activity relationship studies are needed to map the ligand binding site topology on 4R-tau and α-synuclein fibrils, to guide the development of tracers with a higher affinity and selectivity. In addition, deep learning-based drug development such as using AlphaFold or RoseTTaFold and on-chip pharmacokinetics may speed up the development and optimization of imaging tracers (Schneider, 2018; Bhhatarai et al., 2019; Baek et al., 2021; Jumper et al., 2021). Multiscale simulation pipeline combining methods with different accuracy/efficiency such as molecular docking, molecular dynamics simulation, and free energy calculation, will likely provide a high degree of validation of the simulations (Araki et al., 2021).

In summary, amyloid and tau PET imaging have a profound impact on the early and differential diagnosis of dementia and facilitated the development of disease-modifying therapeutics. Further development of 4R tau and α-synuclein specific tracers is needed to fill the unmet need and move toward precision medicine in dementia.

## Author Contributions

RN wrote the draft manuscript. Both authors contributed to the manuscript.

## Conflict of Interest

The authors declare that the research was conducted in the absence of any commercial or financial relationships that could be construed as a potential conflict of interest.

## Publisher’s Note

All claims expressed in this article are solely those of the authors and do not necessarily represent those of their affiliated organizations, or those of the publisher, the editors and the reviewers. Any product that may be evaluated in this article, or claim that may be made by its manufacturer, is not guaranteed or endorsed by the publisher.
